# Influence of tillage practices and straw incorporation on soil aggregates, organic carbon, and crop yields in a rice-wheat rotation system

**DOI:** 10.1038/srep36602

**Published:** 2016-11-04

**Authors:** Ke Song, Jianjun Yang, Yong Xue, Weiguang Lv, Xianqing Zheng, Jianjun Pan

**Affiliations:** 1 College of Natural Resources and Environmental Sciences, Nanjing Agricultural University, Nanjing 210095, China; 2 Institute of Eco-Environment and Plant Protection, Shanghai Academy of Agricultural Sciences, Shanghai 201403, China

## Abstract

In this study, a fixed-site field experiment was conducted to study the influence of different combinations of tillage and straw incorporation management on carbon storage in different-sized soil aggregates and on crop yield after three years of rice-wheat rotation. Compared to conventional tillage, the percentages of >2 mm macroaggregates and water-stable macroaggregates in rice-wheat double-conservation tillage (zero-tillage and straw incorporation) were increased 17.22% and 36.38% in the 0–15 cm soil layer and 28.93% and 66.34% in the 15–30 cm soil layer, respectively. Zero tillage and straw incorporation also increased the mean weight diameter and stability of the soil aggregates. In surface soil (0–15 cm), the maximum proportion of total aggregated carbon was retained with 0.25–0.106 mm aggregates, and rice-wheat double-conservation tillage had the greatest ability to hold the organic carbon (33.64 g kg^−1^). However, different forms occurred at higher levels in the 15–30 cm soil layer under the conventional tillage. In terms of crop yield, the rice grown under conventional tillage and the wheat under zero tillage showed improved equivalent rice yields of 8.77% and 6.17% compared to rice-wheat double-cropping under zero tillage or conventional tillage, respectively.

Globally, soil stores approximately 1,500 Pg of carbon in the form of organic carbon. The soil organic carbon (SOC) pool is 2.5 and 2 times the carbon pool in terrestrial vegetation and the atmosphere, respectively^1^. Thus, small changes in soil organic carbon can cause dramatic changes in the concentration of atmospheric CO_2_^2^. Before this problem was recognized, the primary purpose of soil tillage was to create suitable soil environmental conditions for crop growth, to conserve soil water, and to promote crop-yield increases^3^. However, with the increased awareness of the greenhouse effect, soil organic carbon has been found to be easily affected by tillage and fertilization, and various tillage practices were found to exert a significant influence on soil disturbances, aggregate stability, and organic carbon flux rates^4^,^5^,^6^. Thus, the purpose of soil tillage was no longer limited to increasing crop yields, and a greater consideration was given to the efficacy of enhancing soil carbon preservation and preventing the occurrence of greenhouse effects^7^,^8^.

Extensive studies have shown that compared to conventional tillage, conservation tillage (including zero tillage, minimum tillage, and straw incorporation) can be effective in reducing soil disturbances and soil aggregate structure destruction. Conservation tillage can also slow the turnover rate of macroaggregates, prevent the decomposition of organic carbon by microbes, and extend the storage period of organic carbon in aggregates^9^,^10^,^11^. However, numerous studies hold the opposite view. For example, Carter^12^ and Blanco-Canquia^13^ believe that conservation tillage induces less carbon storage than conventional tillage throughout the soil profile. Thus, whether conservation tillage can increase organic carbon storage in the soil remains an important issue despite extensive studies that have investigated the influence of various tillage practices and field management measures on the content and storage of soil organic carbon^14^,^15^,^16^,^17^. Moreover, previous research has primarily investigated a particular type of crop in dryland or paddy fields^18^,^19^,^20^, and limited research has been conducted under a background of paddy-dryland rotation. The use of any tillage measure affects the soil’s physicochemical properties and crop yields at various levels. Additionally, the long-term application of a single tillage measure results in unfavourable soil conditions for crop growth^21^. Therefore, it is unclear whether conventional tillage or zero tillage and straw incorporation with conservation tillage can satisfy the demand of both ecological and production benefits, namely, increasing the capacity of soil carbon preservation and improving crop yields.

In this study, we chose a rice-wheat double cropping system from a rice-wheat cropping area. A fixed-site field experiment was performed in three consecutive years to achieve the following objectives: (1) to determine the influence of various tillage practices and straw incorporation on the soil aggregate percentage distribution, aggregate stability, and associated organic carbon preservation under rice-wheat rotation; (2) to determine the influence of various tillage practices and straw incorporation on rice-wheat yields and yield components under rice-wheat rotation; and (3) to provide evidence for the development of a reasonable tillage management model in rice-wheat cropping areas.

## Results

### Size composition of the soil aggregates

Table 1 presents the test results of the aggregate size composition in the soil samples collected in November 2013. Under the influence of different tillage practices and straw incorporation measures, the percentage of aggregates of different size classes significantly varied in the topsoil (0–15 cm) and subsurface (15–30 cm) layers. Zero tillage increased the percentage of macroaggregates and water-stable macroaggregates in the soil. In the 0–15 cm soil layer, the mean percentage of A1 was 12.77% higher for the three rice-wheat under zero tillage (RWzt) treatments (T1, T2, and T3) compared to the rice-wheat under conventional tillage (RWct) treatments (T7, T8, and T9). The increase in the percentage of WSA1 was 43.21%. The same trend was observed in the 15–30 cm soil layer. Moreover, the percentage of WSA2 was significantly higher for the RWzt treatment than for the RWct treatment and the treatment of rice under conventional tillage + wheat under zero tillage (RctWzt); the increases were 24.90% and 17.90% in the 0–15 cm soil layer and up to 39.35% and 33.45% in the 15–30 cm soil layer. Under the same tillage practice, the percentage of soil aggregates did not show significant differences among the three treatments of straw incorporation regardless of the depth (0–15 cm or 15–30 cm). However, the percentages of WSA1 and WSA2 in the treatments were ranked as follows: rice-wheat straw incorporation (RWsi) > wheat straw incorporation (Wsi) > no straw incorporation (Nsi). The highest percentages of A1 and WSA1 were observed for the RWzt + RWsi (T3). These high values were higher than those obtained for the RWct + Nsi (T7) by 17.22% and 36.39% in the 0–15 cm soil layer and 28.93% and 66.34% in the 15–30 cm soil layer.

### Soil aggregate-associated organic carbon

As shown in Table 2, regardless of the results obtained by dry or wet sieving, the SOC concentration in the aggregates of various size classes was markedly higher in the 0–15 cm layer compared to that in the 15–30 cm soil layer. Among the various size classes, the SOC concentration in the dry-sieved aggregates was highest for M1 in the 0–15 cm soil layer, which had a mean of 31.08 g·kg^−1^ for the different treatments. In addition, the aggregate-associated SOC generally occurred at similar levels in the other three size classes (A1, A2, and <0.106 mm aggregates (P2)) and had means of 21.28, 22.20, and 20.05 g·kg^−1^, respectively. The SOC concentrations in the water-stable aggregates obtained by wet sieving were ranked as follows: WSA2 > WSM1 > WSA1, with means of 25.14, 23.34, and 20.54 g·kg^−1^. Compared to the 0–15 cm soil layer, limited differences were observed for the aggregate-associated SOC concentrations between various size classes in the 15–30 cm soil layer, and the mean of the different treatments ranged from 10.42 to 11.77 g·kg^−1^.

For the different treatments, the aggregate-associated SOC concentration in the 0–15 cm soil layer ranked higher in the RWzt treatment than in the RctWzt treatment group and RWct treatment group. In addition, the two treatment groups with straw incorporation produced higher aggregate-associated SOC concentrations than the treatment without straw incorporation. The dry sieving results showed that the aggregate-associated SOC concentrations in the A1, A2, and M1 size classes were significantly higher in the RWzt treatment group than in the RWct treatment group (F = 8.265, 5.483, and 10.326, respectively); however, significant differences were not observed between the RctWzt treatment group and the above two groups. The above trend was more obvious in the water-stable aggregates. This result indicates that the double crop under zero tillage was conducive to improving the aggregate-associated SOC concentrations. For the double crop under zero tillage groups, the SOC concentrations of the aggregates of various size classes were significantly higher for the double crop (T3) and single-crop straw incorporation (T2) treatments compared to the no straw incorporation (T1) group regardless of the results obtained by dry or wet sieving. Similarly, the same trend was observed in the RWct and RctWzt treatment groups. In particular, the T3 treatment with RWzt + RWsi resulted in higher SOC concentrations in the aggregates of various size classes compared to the other treatments. According to the results of dry sieving, the aggregate-associated SOC concentrations in the A1, A2, and M1 size classes were higher than those under the T7 treatment with differences of 12.90%, 26.50%, and 17.74%, respectively, which reached statistical significance. The differences between the treatments were more significant in the results obtained by wet sieving. The aggregate-associated SOC concentrations in WSA1, WSA2, and WSM13 were higher under the T3 treatment compared to those under the T7 treatment by 25.04%, 28.55%, and 18.12%, respectively.

In the 15–30 cm soil layer, the aggregate-associated SOC concentration under the different tillage practices presented the following rank: RWct > RctWzt > RWzt, which differed from the trend in the 0–15 cm soil layer. For the three treatments without straw incorporation (T7, T4, and T1), the mean SOC concentrations in the dry-sieved aggregates of the various size classes were 11.22, 10.35, and 10.19 g·kg^−1^, respectively, and in the wet-sieved aggregates, they were 11.60, 10.83, and 10.33 g·kg^−1^, respectively. The mean values obtained by both methods were markedly higher for T7 than for T4 and T1. Under the same tillage practice, straw incorporation increased the aggregate-associated SOC concentrations in the 15–30 cm soil layer. For the three treatments with rice-wheat under conventional tillage (T7, T8, and T9), the total SOC concentrations in the aggregates of various size classes under the dry sieving method were 11.22, 12.42, and 12.44 g·kg^−1^, respectively, whereas the SOC concentrations in water-stable aggregates were 11.60, 12.14, and 12.33 g·kg^−1^, respectively. The values of the T9 treatment with double-crop straw incorporation and the T8 treatment with single-crop straw incorporation were markedly higher than those of the T7 treatment without straw incorporation. Similar trends were observed in the results obtained for the other two tillage practices.

### Crop yields

**Figure 1:**
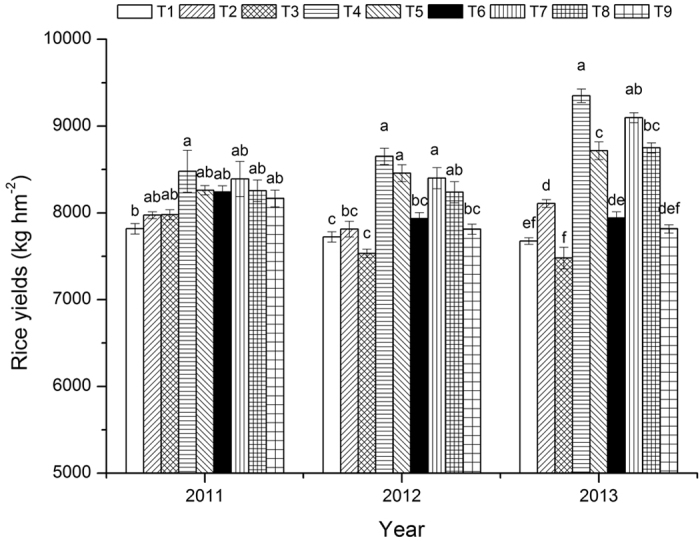
Influence of tillage and straw incorporation on rice yields.

According to the crop yields from the six crop seasons over three years (Fig. 1), the mean rice yield of the nine treatments in 2011 was 8,173.99 kg/hm^2^, and the coefficient of variance (CV) was 3.34%. The highest rice yield was found for the T4 treatment with RctWzt, 8,763.15 kg/hm^2^. The lowest rice yield was found for the T1 treatment with RWzt, 7,699.67 kg/hm^2^. In 2012, the rice yields varied between 7,534.20 and 8,651.60 kg/hm^2^ and presented a mean of 8,063.15 kg/hm^2^ and a CV of 4.84%. There were more significant differences between treatments in 2012 than in 2011. In 2013, the rice yields of the various treatments ranged from 7,232.85 to 9,457.51 kg/hm^2^, the mean was 8326.16 kg/hm^2^, and the CV was 7.82%. Under the different tillage practices, the mean rice yields were 8,669.14 kg/hm^2^ for the RctWzt treatment group and 8,554.94 kg/hm^2^ for the RWct treatment group. These two values were significantly higher than the mean rice yield from the WRzt treatment group (F = 8.206, P < 0.05). This result indicates that conventional tillage was more conducive to increasing rice yields. In particular, the tillage model wherein wheat under zero tillage precedes rice under conventional tillage could increase rice yields. With regard to straw incorporation, the mean rice yields were 8,640.92 and 8,525.47 kg/hm^2^ for the treatment groups of no straw incorporation and wheat straw incorporation + no rice straw incorporation, respectively. These two values were significantly higher than the mean rice yield from the treatment group of double-crop straw incorporation (F = 9.148, P < 0.05). This result indicates that straw incorporation for both crops after the harvest could reduce rice yields.

**Figure 2:**
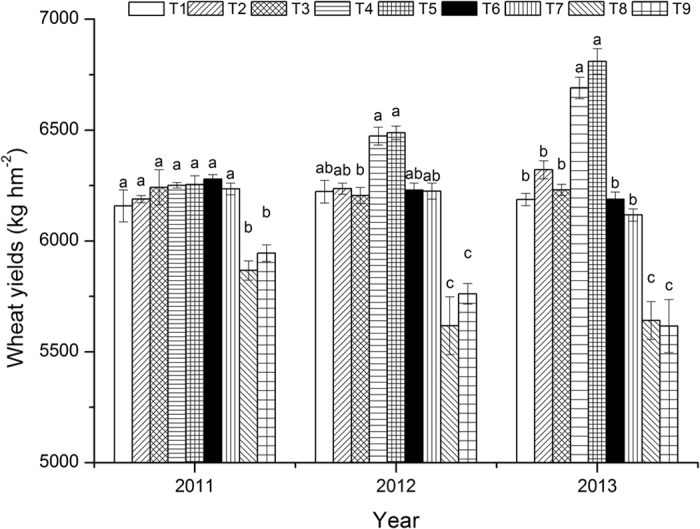
Influence of tillage and straw incorporation on wheat yields.

For wheat (Fig. 2), the yields of the nine treatments ranged from 6,196.45 to 6,289.05 kg/hm^2^ in the year 2011, the mean was 6,248.86 kg/hm^2^, and the CV was 1.14%. In 2012, the wheat yields of the treatments varied between 5,379.75 and 6,546.30 kg/hm^2^, the mean was 6,162.04 kg/hm^2^, and the CV was 4.73%. In 2013, the wheat yields of the various treatments fell in the range of 5,391.06–6,920.18 kg/hm^2^, the mean was 6,200.26 kg/hm^2^, and the CV was 6.37%. A comparison of the tillage treatments showed that from 2012, the wheat yields ranked as follows: RctWzt > RWzt > RWct. In 2013, the mean wheat yields for the above three treatments were 6,562.94, 6,246.13, and 5,791.69 kg/hm^2^, respectively, which showed significant differences between tillage practices (F = 24.015, P < 0.05). This result indicates that sowing rice under zero tillage could improve the following wheat yields. In particular, wheat under zero tillage + rice under conventional tillage resulted in the highest wheat yield in the following harvest, whereas rice under conventional tillage caused a yield reduction in the following wheat harvest. With respect to straw incorporation, the following trend was found in wheat yields under the same tillage practice from 2012: Nsi > Wsi > RWsi. In 2013, however, the mean wheat yields for the above three treatments were 6,331.44, 6,257.57, and 6,011.75 kg/hm^2^, respectively, which showed no significant differences.

### Crop yield components

The results of the rice yield components in 2013 (Table 3) showed that different tillage practices primarily affected the effective panicle number and grain number per panicle in rice. The results of the ANOVA showed that effective panicle number was higher for the RctWzt and RWct treatment groups than for the RWzt treatment group. The differences among the above treatment groups was statistically significant (F = 5.633, P < 0.05). The grain number per panicle showed a similar trend. The mean grain number per panicle was highest for the treatment group of RWct and lowest for the treatment group of RWzt. There was a significant difference between the above two treatments (F = 5.372, P < 0.05). The results indicate that conventional tillage could increase the effective panicle number and grain number per panicle in rice, which were the two main factors leading to increases in rice yields. The analysis of straw incorporation revealed that the effective panicle number was markedly lower with double-crop straw incorporation compared to no straw incorporation, and the difference was statistically significant (F = 2.710, P < 0.05). However, significant differences were not obtained between wheat straw incorporation and the above two treatments. Moreover, the different straw incorporation measures caused no significant differences in the grain number per panicle, 1000-grain weight, or seed setting rate. This result indicates that excessive straw incorporation in two consecutive crop seasons was not favourable for straw decomposition and mineralization. This measure could affect the emergence or growth of rice seedlings, thereby resulting in a reduction in effective panicle number and crop yields.

The results of the wheat yield components in 2013 (Table 4) showed that zero tillage increased the effective panicle number in wheat. The results of the ANOVA revealed that the mean effective panicle numbers of wheat were 669.49 × 10^4^/hm^2^ for the RctWzt treatment group and 635.64 × 10^4^/hm^2^ for the RWzt treatment group. These two values were significantly higher than that for the RWct treatment group, which was 573.70 × 10^4^/hm^2^ (F = 13.705, P < 0.05). Except for the difference in effective panicle number, various tillage practices did not cause significant differences in the parameters of wheat yield components, including the grain number per panicle, 1000-grain weight, and seed setting rate. The analysis of various straw incorporation measures showed that the effective panicle number of wheat was relatively high for the treatment group with wheat straw incorporation; however, various straw incorporation rates did not present significant differences in the parameters of wheat yield components. These results indicate that zero tillage could increase the effective panicle number in wheat, which was the primary factor leading to an increase in wheat yields.

## Discussion

**Figure 3:**
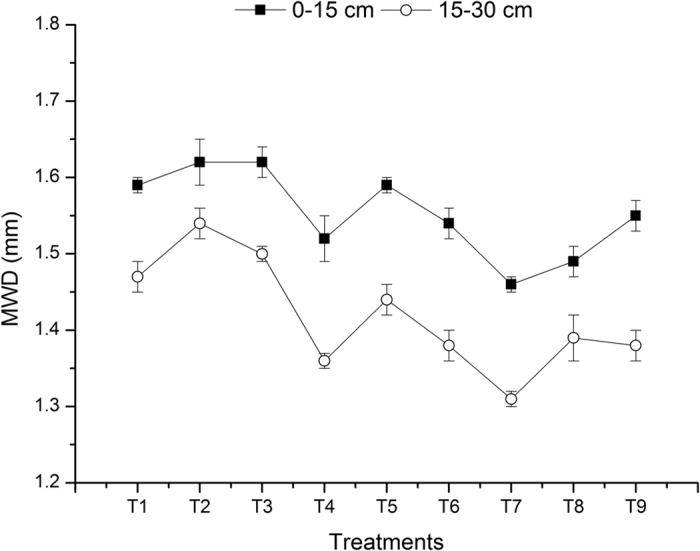
Mean weight diameter (MWD) of soil aggregates.

**Figure 4:**
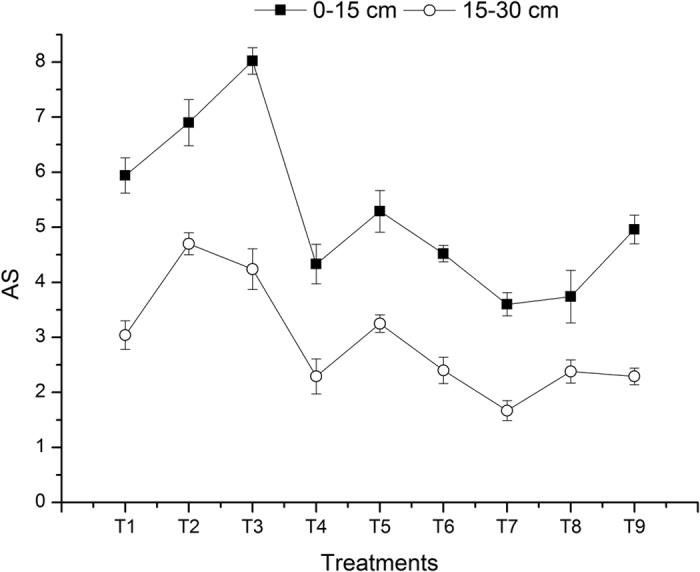
Soil aggregate stability (AS).

The management measures that include tillage and straw incorporation not only determine land productivity but also affect soil microbial biomass and activity by altering the temperature and humidity of the soil, the growth stage of the roots, and the quantity and quality of the crop residues, ultimately affecting the content and stability of soil aggregates^22^,^23^,^24^. Figures 3 and 4 show that both the mean weight diameter (MWD) and aggregate stability (AS) of the soil aggregates were higher for the RWzt + RWsi treatment than for the RWzt + Nsi treatment. From the perspective of a farmland ecosystem, zero tillage and straw incorporation enable the topsoil to form a complex decomposition sub-system that simulates the natural ecosystem. This sub-system can buffer the impact of external force on the soil mass and gather matter and energy in the topsoil under zero tillage, where the crop roots are growing. This phenomenon of identical distributions can improve the nutrient recycling capacity and energy utilization efficiency^25^,^26^,^27^. However, the input of plant residues combined with zero tillage can protect the topsoil from frequent wet-dry freeze-thaw effects, thereby increasing the content of stable macroaggregates and reducing the mineralization rate of soil organic carbon^28^. Our results showed that the mean percentages of >2 mm macroaggregates and water-stable macroaggregates were increased by 12.77% and 43.21%, respectively, for the treatment group of rice-wheat under zero tillage compared to RWct. In the 0–15 cm and 15–30 cm soil layers, the percentage of 2–0.25 mm water-stable macroaggregates was increased by 25% and 40%, respectively, for the RWzt treatment compared to the RWct treatment. Thus, compared to conventional tillage, zero tillage can reduce the turnover of macroaggregates in farmland and facilitate the enclosure of organic carbon in microaggregates, which enables microaggregates to preserve more physically protected organic carbon and form more macroaggregates^29^. Moreover, our results showed that under the same tillage practice, the treatment of RWsi resulted in a 10–40% higher percentage of macroaggregates and water-stable macroaggregates relative to the treatment without straw incorporation. This result is mainly because fresh straw and other organic materials release organic substances such as polysaccharides and organic acids. These organic substances provide attachment points and nutrients for the growth of fungi and microorganisms while simultaneously promoting the cementation of straw and soil particles to form macroaggregates^30^. Fonte *et al*.^31^ propose that a massive input of plant residues and the avoidance of disturbance under zero tillage are the main factors underlying the improved content and stability of macroaggregates in the surface soil layer.

**Figure 5:**
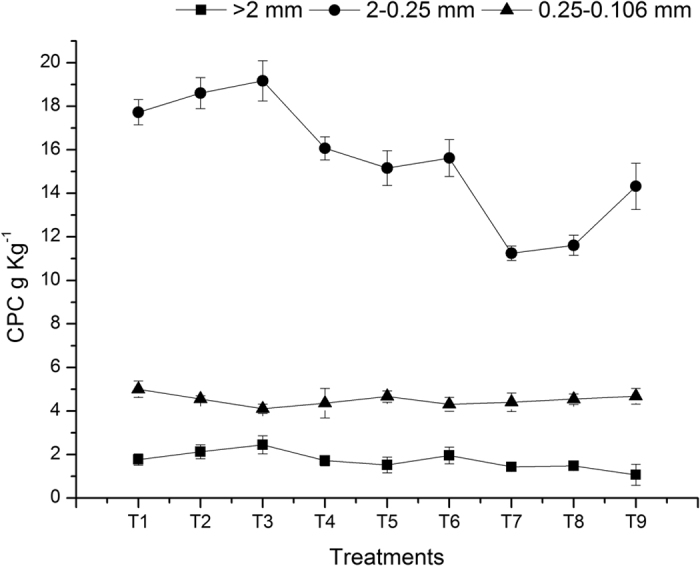
Influence of treatments on the carbon preservation capacity of different soil aggregates (0–15 cm).

**Figure 6:**
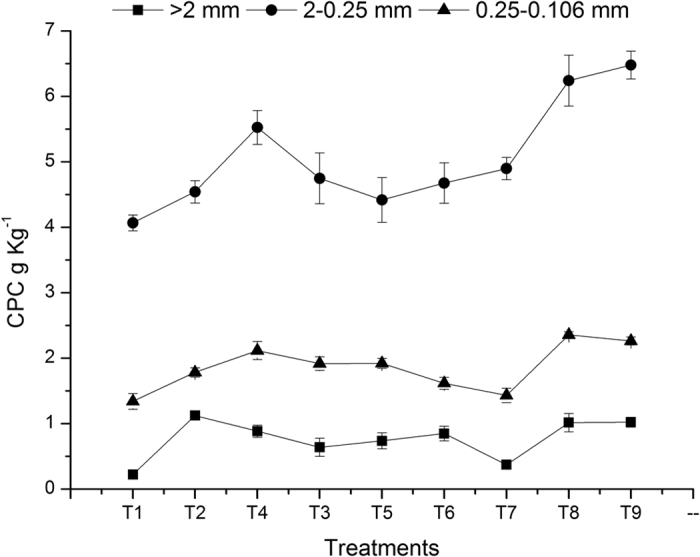
Influence of treatments on the carbon preservation capacity of different soil aggregates (15–30 cm).

Regarding SOC protection by soil aggregates, the newly added carbon provides physical protection and is then subjected to chemical conversion and structural stabilization; meanwhile, alternation of the properties and distribution of the carbon pool leads to both the diversification of aggregate-scale microbial habitats and the evolution of microbial biota, along with changes in various fertility service functions such as functional groups and enzyme activity, which promote the development of diverse biota and thereby stabilize ecosystem processes. Thus, the protective mechanism of aggregates for SOC can be summarized as three interacting and interdependent processes: physical protection, chemical bonding and stabilization, and adaptation of microbial biota and functional groups^32^. The above results showed that zero tillage resulted in higher organic carbon storage in soil aggregates in the 0–15 cm soil layer than did conventional tillage (Figs 5 and 6), primarily because conservation tillage reduces the damage to soil aggregates and increases the content and stability of associated organic carbon accordingly. Similar findings were reported by Srinivasan *et al*.^33^ and Six *et al*.^34^, who proposed that minimum tillage results in higher SOC content in the 0–15 cm soil layer compared to conventional tillage because of the lower contents of organic carbon and water-stable aggregates in the soil under conventional tillage. Our results also showed that regardless of tillage practice, the highest SOC concentration was found for the 0.25–0.106 mm microaggregates in the 0–15 cm and 15–30 cm soil layers, which is inconsistent with the result of Six *et al*.^15^, who found that >2 mm aggregates had the highest SOC level compared to the other size classes of aggregates. Six *et al*.^15^ suggested that macroaggregates are formed by the aggregation of soil particles through cementation of organic substances and indicated that macroaggregate particles are the main carrier of organic carbon. In the present study, light-fraction organic carbon was not deducted from the aggregate components, which largely increased the SOC concentration in the 0.25–0.106 mm aggregates. Moreover, it has been suggested that microaggregates, which possess a larger specific surface area with more abundant active points, can adsorb organic substances and preserve organic carbon through strong ligand exchange and multivalent cation bridging. Consequently, the SOC levels are even higher in microaggregates than in macroaggregates^35^,^36^.

Numerous studies focusing on the influence of tillage on soil organic carbon have only considered the shallow soil layer (0–15 cm) and ignore the SOC level in the deep soil if it shows little differences or exhibits the same distribution as observed in the shallow soil^37^,^38^,^39^,^40^. In the present study, the SOC concentrations of various treatments showed inconsistent trends between the 15–30 cm and 0–15 cm soil layers. In the 0–15 cm soil layer, the RWzt treatment resulted in a higher percentage of soil aggregates than did the RWct treatment. In addition, the aggregate-associated SOC concentration was higher for RWzt. In the 15–30 cm soil layer, the percentage of soil aggregates did not show ordered differences; however, the SOC concentration was higher with conventional tillage than with conservation tillage, exhibiting the following rank: RWct > RctWzt > RWzt. This result indicates that it is necessary to consider deeper soil when assessing the influence of tillage practices on soil organic carbon. Particularly in farmland under rice-wheat rotation, different tillage practices combined with the alternating wet and dry soil environment cause quantitative changes in the soil organic carbon of deep soil. However, compared to zero tillage, conventional tillage, such as ploughing, incorporates organic materials, including straw mulch at the soil surface and residual roots in shallow soil, into deeper soil. The organic materials are tightly bound to soil particles, thereby improving the stability of their mineralization and promoting the accumulation of organic carbon in the deep soil^41^. This result also indicates that the analysis of organic carbon only in shallow soil will result in underestimated values. Moreover, the influence of different farmland management practices (e.g., tillage and straw incorporation) on organic carbon in the deep soil must be further investigated to appropriately estimate the carbon preservation potential of bulk soil, particularly in deep soil.

Extensive studies have shown that zero tillage can improve wheat yields^42^,^43^. However, different results have been obtained for the effect of zero tillage on rice yields, including yield improvement and yield reduction. There are different opinions about the reason for rice yield reduction. Zhuang^44^ suggested that zero tillage results in a higher bulk density in the 7–14 cm soil layer, which might be the primary cause of early ageing and yield reduction in rice. Feng^45^ reported that rice yield reductions might be attributable to the enhancement of soil permeability under zero tillage, which accelerates water percolation and fertilizer leakage, resulting in decreased rice tillering and inadequate population. In the present study, zero tillage markedly improved wheat yields. More specifically, the yield increase reached 13.32% for RctWzt compared to RWct. For rice, however, zero tillage produced a negative effect and caused a yield reduction of 10.55%. The highest crop yield of the entire wheat-rice rotation was obtained for the model of RctWzt. Combined with the above results of the aggregate-associated SOC and yield components, we conclude that zero tillage can cause nutrient enrichment in the topsoil^46^,^47^,^48^, increase the contents of soil aggregates and organic carbon, and thus increase the effective panicle number and improve the yield of wheat. However, tillage is not applied to paddy fields under zero tillage; thus, it is difficult to blend the soil and fertilizers. At the early growth stage of rice, substantial nutrients are lost via runoff or evaporation in the topsoil, resulting in lower levels of soil-available nutrients at the mature stage of rice, which in turn causes a shortage of fertilizers at the middle to late stage of rice. Consequently, the effective panicle number and grain number per panicle were both lower for rice under zero tillage than for rice under conventional tillage. This may be the primary cause of rice yield reductions under zero tillage. Therefore, it is recommended to grow wheat under zero tillage and rice under conventional tillage in rice-wheat cropping areas, thus achieving a high yield and saving labour.

It is indisputable that straw incorporation can improve soil fertility, reduce soil bulk density, enhance soil permeability, and increase organic carbon content in the soil^49^. Our results also showed that with increases in the straw incorporation rate, the soil carbon preservation capacity (CPC) was enhanced and the aggregate-associated SOC concentration was increased. However, in terms of crop yield, straw incorporation might affect wheat yields. In particular, rice-wheat straw incorporation caused a reduction in wheat yields, primarily because excess incorporated straw cannot be rapidly decomposed and mineralized. After entering the surface soil layer and mixing with the soil through conventional tillage, the large spacing among straw pieces in the topsoil causes serious soil water losses and affects seedling germination and rooting. Water shortages and straw obstacles affect the emergence rate of wheat and growth quality at the seedling stage. Consequently, wheat yields were markedly reduced under straw incorporation combined with conventional tillage. In the paddy fields, because there is an adequate water supply and a strong water retention capacity, an appropriate rate of straw incorporation will not affect the growth of rice seedlings. However, excessive straw incorporation continues to affect the effective panicle number of rice, which is unfavourable for a high yield. Additionally, the process of straw decomposition and mineralization is associated with the release of toxins, which can prevent the growth and development of crop seedlings and further affect crop yields.

## Conclusions

Our results demonstrated that zero tillage and straw incorporation increased the contents of soil macroaggregates and organic carbon under a rice-wheat double cropping system. After three years of rice-wheat rotation, zero tillage and straw incorporation in rice-wheat double-cropping significantly increased (compared to conversion tillage) the percentage of >2 mm macroaggregates and water-stable macroaggregates and aggregate- and water stable aggregate-associated organic carbon in the soil. They also increased the mean weight diameter and stability of the soil aggregates. However, in the straw incorporation and conventional tillage treatments, straw mulch on the soil surface and residual roots from the shallow soil were incorporated by ploughing into the deep soil layer, and different forms of organic carbon occurred at higher levels in the 15–30 cm soil layer under the conventional tillage system compared to the zero tillage system. In terms of crop yield, the rice grown under the conventional tillage and the wheat grown under zero tillage showed improved equivalent rice yields of 8.77% and 6.17% compared to the rice-wheat double-cropping under zero tillage and conventional tillage, respectively. Based on the ecological and yields dual benefits, growing wheat under zero tillage and rice under conventional tillage are recommended in combination with single-crop straw incorporation in the rice-wheat cropping area.

## Materials and methods

### Study area

The study was conducted on the Changjiang Farm (121°33′05″, 31°42′10″) in Chongming County, Shanghai, China. The study area is located in the northern subtropical zone under a typical subtropical monsoon climate. The annual average temperature is 15 °C, the annual average rainfall is 1,003.7 mm, and the annual average sunshine time is 2,104.0 hours. The frost-free period lasts approximately 229 days. The experimental soil was a waterloggogenic paddy soil. The topsoil contained organic matter, 14.0 g·kg^−1^; total nitrogen, 0.85 g·kg^−1^; alkali-hydrolysable nitrogen, 82.0 mg·kg^−1^; available phosphorus, 41.1 mg·kg^−1^; and available potassium, 118.3 mg·kg^−1^. The pH was 8.2 (water:soil ratio = 5:1). The cropping system under study was a rice-wheat rotation.

### Experimental materials

In the experiment, rice (*Oryza sativa* L.) cultivar Hanyou 8 was planted at a seeding rate of 185 kg·hm^−2^ and a row spacing of 23 cm. Wheat (*Triticum aestivum*) cultivar Wanmai 52 was planted at a seeding rate of 120 kg·hm^−2^ and a row spacing of 25 cm. Straw incorporation was performed during the wheat-growing season using rice straw (C/N = 51) harvested from the experimental plots’ preceding crop, which contained 38.7% cellulose, 21.7% hemicellulose, and 19.3% lignin. Straw incorporation was performed during the rice-growing season using wheat straw (C/N = 82.74) harvested from the preceding crop, which contained 35.72% cellulose, 21.93% hemicellulose, and 18.64% lignin. Nitrogen, phosphorous, and potassium fertilizers were applied as urea (N-46%), calcium superphosphate (P_2_O_5_-46%), and potassium chloride (K_2_O-60%), respectively.

### Experimental design and field management

The experiment started in October 2010. Nine treatments were set up with different tillage practices (zero tillage and conventional plough tillage) and straw incorporation rates (Table 5).

Among these treatments, conventional tillage involved ploughing to a depth of 30 cm. There were three replicates per treatment, which were arranged using a single-factor randomized block design. Each plot was 40 m long and 6 m wide and covered an area of 240 m^2^. The ridges between plots and irrigation ditch ridges between blocks were wrapped and separated using plastic film. Fertilizers were applied at the following rates: N, 225 kg·hm^−2^; P_2_O_5_, 90 kg·hm^−2^; and K_2_O, 90 kg·hm^−2^ for rice; N, 270 kg·hm^−2^; P_2_O_5_, 60 kg·hm^−2^; and K_2_O, 90 kg·hm^−2^ for wheat. Straw incorporation was performed at a 100% rate (straw was pulverised into 10 cm lengths at harvest using an automatic harvester). For each crop, pulverised straw, phosphorous fertilizer, and potassium fertilizer were applied once as basal dressing before sowing. Nitrogen fertilizer was applied in two parts as basal and top dressings. The basal dressing of nitrogen fertilizer was applied at 135 and 162 kg·hm^−2^ for rice and wheat, respectively. The remainder of the nitrogen fertilizer was applied as a top dressing. Other field management and pest control followed local conventional practices.

### Sample collection and analysis

Before the start of the experiment, basic soil samples were collected in September 2010. After the start of the experiment, rice and wheat yields were analysed in June 2011, June 2012, and June 2013 following the wheat harvest and in November 2011, November 2012, and November 2013 following the rice harvest. The soil samples were collected in November 2013. A soil auger was used to collect samples to depths of 0–15 cm and 15–30 cm in an “S” pattern. The soil samples were immediately transported to the laboratory. Animal and plant debris, along with stones and other debris, were removed before use^50^. The size composition of the soil aggregates was determined by dry sieving, and the size composition of the water-stable aggregates was analysed using a DIK-2001 soil aggregate analyser (RKC instrument Inc., Saitama-ken, Japan). The aggregate-associated carbon was determined using a CHN-440 (United Instrument Co., Ltd, Chicago, USA) elemental analyser.

### Data calculation and analysis

#### Data calculation


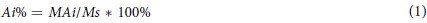


Where *Ai%* is the mass percentage of the aggregates for a certain size class, *MA*_*i*_ is the mass of the aggregates (>2.0 mm, 2–0.25 mm, 0.25–0.106 mm, or <0.106 mm) and *Ms* is the mass of the air-dried soil.


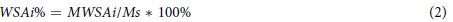


Where *WSAi%* is the mass percentage of the water-stable aggregates for a certain size class, *MWSA*_*i*_ is the mass of the water-stable aggregates in the size class of >2.0 mm, 2–0.25 mm, or 0.25–0.106 mm and *Ms* is the mass of the air-dried soil.


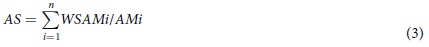


Where *AS* is the aggregate stability of the soil, n = 3, *WSAM*_*i*_ is the mass of water-stable aggregates of >2.0 mm, 2–0.25 mm, or 0.25–0.106 mm and *AM*_*i*_ is the mass of the aggregates of >2.0 mm, 2–0.25 mm, or 0.25–0.106 mm.


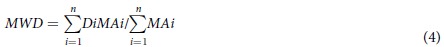


Where *MWD* is the mean weight diameter of the soil aggregates, n = 4, *D*_*i*_ is the mean diameter of the soil particles in each size class (2.0 mm, 1.125 mm, 0.178 mm, or 0.53 mm) and *MA*_*i*_ is the mass of the soil particles (>2.0 mm, 2–0.25 mm, 0.25–0.106 mm, or <0.106 mm).


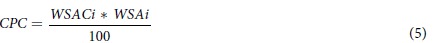


Where *CPC* is the carbon preservation capacity of the soil aggregates, *WSAC*_*i*_ is the concentration of soil carbon in the water-stable aggregates of >2.0 mm, 2–0.25 mm, or 0.25–0.106 mm and *WSA*_*i*_ is the percentage of water-stable aggregates of 2.0 mm, 2–0.25 mm, or 0.25–0.106 mm.


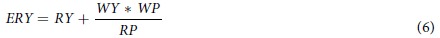


Where *ERY* is the equivalent rice yield, *RY* is the rice yield, *WY* is the wheat yield, *RP* is the government purchase price of rice for the year of the study and *WP* is the government purchase price of wheat for the year of the study.

#### Statistical analysis

The data were processed using Microsoft Excel 2010 (Microsoft Corp., Redmond, WA, USA) and then subjected to an analysis of variance (ANOVA) using the SPSS 17.0 Statistical Package (SPSS Inc., Chicago, IL, USA). Multiple comparisons of various treatments were conducted using Tukey’s method. The graphics were produced using Origin 8.0 (OriginLab Corp., Northampton, MA, USA).

## Additional Information

**How to cite this article**: Song, K. *et al*. Influence of tillage practices and straw incorporation on soil aggregates, organic carbon, and crop yields in a rice-wheat rotation system. *Sci. Rep*. **6**, 36602; doi: 10.1038/srep36602 (2016).

**Publisher’s note:** Springer Nature remains neutral with regard to jurisdictional claims in published maps and institutional affiliations.

## Figures and Tables

**Table 1:** Influence of tillage and straw incorporation on the size composition of soil aggregates.

Treatments	A1 (%)	A2 (%)	M1 (%)	P2 (%)	WSA1 (%)	WSA2 (%)	WSM1 (%)
0–15 cm depth\size class	>2 mm	2–0.25 mm	0.25–0.106 mm	<0.106 mm	>2 mm	2–0.25 mm	0.25–0.106 mm
T1	61.70 ± 1.85a	30.91 ± 0.63c	5.93 ± 0.66c	1.46 ± 0.03c	8.46 ± 0.74bc	63.07 ± 0.46ab	18.43 ± 0.46bcd
T2	60.83 ± 5.43ab	35.32 ± 1.18a	3.02 ± 1.17d	0.83 ± 0.01d	9.72 ± 2.77ab	66.78 ± 0.62a	18.04 ± 0.54bcd
T3	61.82 ± 2.84a	33.67 ± 0.15ab	3.46 ± 1.84d	1.05 ± 0.05cd	10.42 ± 3.69a	67.47 ± 3.20a	16.35 ± 0.04d
T4	57.58 ± 3.58abc	32.84 ± 0.27bc	7.74 ± 0.20bc	1.84 ± 0.02b	7.55 ± 2.41c	59.85 ± 1.18bc	19.88 ± 1.83bc
T5	59.22 ± 0.92ab	35.32 ± 0.92a	4.62 ± 1.13cd	0.84 ± 0.01d	9.57 ± 1.81b	62.07 ± 1.67abc	18.59 ± 2.12bcd
T6	56.37 ± 2.29bc	33.63 ± 3.36ab	8.07 ± 1.72b	1.93 ± 0.03ab	9.61 ± 1.83ab	45.44 ± 0.19d	22.64 ± 0.19a
T7	52.74 ± 1.03c	34.22 ± 1.62a	10.52 ± 3.24a	2.52 ± 0.16a	7.64 ± 4.30c	50.88 ± 1.49c	20.73 ± 1.30bc
T8	53.47 ± 1.72c	35.64 ± 0.73a	8.84 ± 0.86b	2.05 ± 0.07ab	7.05 ± 2.77c	49.54 ± 0.85c	19.64 ± 1.19bc
T9	57.26 ± 0.43abc	34.92 ± 0.64a	6.35 ± 1.05bc	1.47 ± 0.10c	5.28 ± 0.48d	57.55 ± 1.05bc	20.45 ± 0.20bc
**Mean**	**57.89**	**34.05**	**6.51**	**1.55**	**8.36**	**58.07**	**19.42**
15–30 cm depth\size class	>2 mm	2–0.25 mm	0.25–0.106 mm	<0.106 mm	>2 mm	2–0.25 mm	0.25–0.106 mm
T1	55.73 ± 0.93bc	26.96 ± 0.82c	13.79 ± 0.64c	3.52 ± 0.67a	8.47 ± 1.49b	49.42 ± 2.57bc	17.48 ± 1.31b
T2	56.48 ± 3.73ab	31.67 ± 1.32ab	8.84 ± 0.80d	3.01 ± 2.01a	10.04 ± 0.82a	55.77 ± 1.86a	20.35 ± 1.81a
T3	58.52 ± 0.64a	31.57 ± 1.77ab	7.16 ± 3.23d	2.75 ± 1.84a	10.18 ± 1.74a	56.48 ± 1.18a	19.74 ± 0.66a
T4	49.51 ± 4.16d	32.13 ± 0.25a	15.30 ± 1.81bc	3.06 ± 0.18a	6.77 ± 0.66c	38.64 ± 2.60c	16.22 ± 1.20b
T5	53.48 ± 4.85c	33.90 ± 1.61a	9.66 ± 1.67d	2.96 ± 0.80a	8.63 ± 1.48b	46.20 ± 0.55b	14.63 ± 0.40c
T6	52.66 ± 1.23c	27.55 ± 3.20c	16.53 ± 1.94b	3.26 ± 0.06a	9.56 ± 0.28ab	36.31 ± 0.29c	14.63 ± 0.48c
T7	45.39 ± 1.71e	32.40 ± 1.19a	18.47 ± 1.05a	3.74 ± 0.37a	6.12 ± 0.08c	33.22 ± 0.84d	11.12 ± 0.24d
T8	48.74 ± 1.25d	30.98 ± 0.84abc	16.84 ± 0.29b	3.44 ± 0.76a	6.58 ± 1.18c	45.34 ± 1.67b	12.64 ± 1.82d
T9	49.8 ± 0.07d	31.79 ± 0.73ab	15.43 ± 0.85bc	2.98 ± 0.14a	6.34 ± 0.64c	37.46 ± 0.85c	15.49 ± 1.17bc
**Mean**	**52.26**	**30.99**	**13.56**	**3.19**	**7.07**	**44.32**	**15.81**

**Table 2:** Influence of tillage and straw incorporation on soil aggregate-associated organic carbon concentrations.

Treatments	Aggregate-associated SOC (g kg^−1^ soil aggregate)						
(0–15 cm)	A1-C	A2-C	M1-C	P2-C	WSA1-C	WSA2-C	WSM1-C
	>2 mm	2–0.25 mm	0.25–0.106 mm	<0.106 mm	>2 mm	2–0.25 mm	0.25–0.106 mm
T1	21.56 ± 0.27abc	22.36 ± 0.37bc	31.68 ± 0.24bc	20.04 ± 2.14abc	20.28 ± 0.58bc	24.89 ± 0.24bc	22.89 ± 1.04bc
T2	22.46 ± 0.35a	22.51 ± 0.23bc	33.64 ± 0.64a	21.04 ± 0.08a	21.86 ± 0.17b	27.86 ± 1.25a	25.24 ± 0.46a
T3	22.40 ± 0.58a	25.44 ± 0.81a	33.52 ± 0.21a	19.79 ± 1.43bc	23.47 ± 2.47a	28.41 ± 0.12a	25.10 ± 0.58a
T4	20.46 ± 0.87bc	20.38 ± 0.73d	28.44 ± 1.59d	18.78 ± 0.12c	18.43 ± 0.07c	23.60 ± 0.37bc	22.11 ± 1.84bc
T5	21.74 ± 1.24ab	23.13 ± 2.37bc	29.32 ± 2.13cd	20.74 ± 0.09abc	20.35 ± 0.28bc	25.48 ± 1.10abc	23.64 ± 2.32b
T6	21.90 ± 2.31ab	21.34 ± 0.11bcd	30.80 ± 0.68cd	18.90 ± 0.11c	20.47 ± 0.80bc	25.17 ± 0.73ab	23.17 ± 0.12b
T7	19.84 ± 0.32c	20.11 ± 1.50d	28.47 ± 0.09d	19.52 ± 1.42bc	18.77 ± 0.06c	22.10 ± 0.49c	21.25 ± 0.80c
T8	20.91 ± 0.15bc	21.67 ± 0.23bcd	32.25 ± 0.38abc	21.48 ± 0.85a	21.05 ± 0.92b	23.44 ± 0.12bc	23.16 ± 0.35b
T9	21.21 ± 0.08abc	22.86 ± 0.48bc	31.60 ± 0.91bc	20.12 ± 0.87abc	20.17 ± 1.09bc	25.33 ± 0.86abc	23.48 ± 0.35b
Mean	**21.28**	**22.2**	**31.08**	**20.05**	**20.54**	**25.14**	**23.34**
(15–30 cm)	A1-C	A2-C	M1-C	P2-C	WSA1-C	WSA2-C	WSM1-C
	>2 mm	2–0.25 mm	0.25–0.106 mm	<0.106 mm	>2 mm	2–0.25 mm	0.25–0.106 mm
T1	10.58 ± 0.32c	10.18 ± 0.49c	10.36 ± 0.55b	9.64 ± 0.00b	9.83 ± 0.46b	10.12 ± 0.80c	11.05 ± 0.57a
T2	10.19 ± 0.15c	11.62 ± 0.12bc	11.18 ± 0.12b	11.28 ± 0.24a	11.12 ± 0.12a	11.47 ± 0.12ab	11.46 ± 0.23a
T3	11.39 ± 0.27abc	11.27 ± 0.24bc	11.52 ± 0.37b	10.34 ± 0.12ab	10.10 ± 0.23a	11.19 ± 0.35ab	11.58 ± 0.23a
T4	10.38 ± 0.87c	10.46 ± 0.37c	10.29 ± 0.07b	10.26 ± 0.12ab	10.38 ± 0.23a	10.80 ± 0.12bc	11.31 ± 0.12a
T5	11.22 ± 0.24abc	11.27 ± 0.25bc	12.25 ± 0.73ab	10.28 ± 0.23ab	10.42 ± 0.23a	11.18 ± 0.11ab	12.10 ± 0.18a
T6	11.24 ± 0.12abc	12.06 ± 0.86ab	11.65 ± 0.07b	10.42 ± 1.04ab	10.87 ± 0.35a	11.43 ± 0.12ab	11.84 ± 0.58a
T7	11.67 ± 0.23ab	11.44 ± 0.73bc	11.64 ± 0.20b	10.11 ± 0.38ab	10.52 ± 0.58a	12.24 ± 0.37a	12.05 ± 0.23a
T8	12.58 ± 0.58a	13.48 ± 1.10a	13.38 ± 0.24a	10.24 ± 0.58ab	11.74 ± 0.12a	12.51 ± 0.32a	12.18 ± 0.09a
T9	12.34 ± 0.42a	12.62 ± 0.12ab	13.58 ± 0.13a	11.21 ± 0.33a	11.95 ± 0.27a	12.67 ± 0.12a	12.37 ± 0.58a
**Mean**	**11.29**	**11.6**	**11.76**	**10.42**	**10.77**	**11.51**	**11.77**

**Table 3:** Influence of tillage and straw incorporation on the rice yield components.

Treatments	Rice			
	Effective panicle number × 10^4^·hm^−2^	Grain number per panicle	1000-Grain weight (g)	Seed setting rate (%)
T1	310.25 ± 1.76c	112.22 ± 1.04ab	25.90 ± 0.22a	93.88 ± 0.24a
T2	319.27 ± 1.18bc	112.38 ± 1.05ab	25.59 ± 0.28a	93.66 ± 0.37a
T3	308.49 ± 3.20c	108.64 ± 0.75c	25.77 ± 0.19a	93.92 ± 0.22a
T4	335.51 ± 1.99a	116.38 ± 2.28a	25.22 ± 0.25a	93.64 ± 0.37a
T5	318.43 ± 2.93bc	114.37 ± 1.13ab	25.71 ± 0.38a	94.72 ± 0.16a
T6	321.22 ± 1.94bc	112.57 ± 2.10ab	25.90 ± 0.37a	93.54 ± 0.514a
T7	334.24 ± 2.25a	117.46 ± 0.87a	25.15 ± 0.10a	94.21 ± 0.28a
T8	322.60 ± 3.11b	114.25 ± 1.24ab	25.42 ± 0.14a	94.23 ± 0.21a
T9	314.46 ± 6.85bc	114.40 ± 2.26ab	25.51 ± 0.11a	93.45 ± 0.21a

**Table 4:** Influence of tillage and straw incorporation on wheat yield components.

Treatments	Wheat			
	Effective panicle number × 10^4^·hm^−2^	Grain number per panicle	1000-Grain weight (g)	Seed setting rate (%)
T1	602.18 ± 8.11bcd	33.20 ± 1.56a	41.38 ± 0.32a	92.56 ± 1.24a
T2	665.12 ± 9.11ab	33.12 ± 0.24a	41.22 ± 0.54a	91.82 ± 1.56a
T3	639.61 ± 9.57abc	31.38 ± 2.14a	40.78 ± 0.28a	91.79 ± 1.28a
T4	687.52 ± 27.53a	32.24 ± 0.37a	41.21 ± 0.21a	91.84 ± 2.31a
T5	712.34 ± 14.26a	32.36 ± 0.58a	41.53 ± 0.42a	92.68 ± 0.89a
T6	608.62 ± 11.96bcd	32.44 ± 0.35a	40.35 ± 0.78a	92.25 ± 0.65a
T7	589.37 ± 19.70cd	31.52 ± 1.55a	40.01 ± 1.23a	90.62 ± 1.14a
T8	567.52 ± 16.04cd	31.22 ± 1.24a	41.51 ± 0.95a	91.87 ± 1.20a
T9	564.24 ± 9.22d	31.14 ± 1.12a	40.74 ± 1.24a	90.86 ± 1.15a

**Table 5:** Tillage and straw incorporation regimens.

Treatments	Items	Abbreviations
T1	Rice-Wheat under zero tillage + No straw incorporation	RWzt + Nsi
T2	Rice-Wheat under zero tillage + Wheat straw incorporation	RWzt + Wsi
T3	Rice-Wheat under zero tillage + Rice-Wheat straw incorporation	RWzt + RWsi
T4	Rice under conventional tillage + Wheat under zero tillage + No straw incorporation	RctWzt + Nsi
T5	Rice under conventional tillage + Wheat under zero tillage + Wheat straw incorporation	RctWzt + Wsi
T6	Rice under conventional tillage + Wheat under zero tillage + Rice-Wheat straw incorporation	RctWzt + RWsi
T7	Rice-Wheat under conventional tillage + No straw incorporation	RWct + Nsi
T8	Rice-Wheat under conventional tillage + Wheat straw incorporation	RWct + Wsi
T9	Rice-Wheat under conventional tillage + Rice-Wheat straw incorporation	RWct + RWsi
